# Letter to the editor regarding ‘the association between nutritional-inflammatory status and chronic kidney disease prognosis: a population-based study’

**DOI:** 10.1080/0886022X.2025.2508292

**Published:** 2025-05-25

**Authors:** Ailing Chen, Ningxu Li

**Affiliations:** Department of Nephrology, Liyuan Hospital, Tongji Medical College, Huazhong University of Science and Technology, Wuhan, Hubei, China

Dear Editor,

We were intrigued by the article by Zhang et al. [1] published in your journal Renal Failure, titled ‘the association between nutritional-inflammatory status and chronic kidney disease prognosis: a population-based study’. Using data from the National Health and Nutrition Examination Survey (NHANES), the authors confirmed that the advanced lung cancer inflammation index (ALI) was positively associated with estimated glomerular filtration rate (eGFR) in patients with chronic kidney disease (CKD) and negatively associated with their all-cause mortality. Subgroup analyses revealed a stronger positive correlation between ALI and eGFR in patients with CKD stages III–V compared to those with stages I–II. However, the protective effect of ALI on mortality was weaker in stages III–V. We commend the authors for their work and agree with their conclusions; however, several issues may impact the interpretation of the data and warrant our attention.

Firstly, the method of variable selection for multivariate Cox regression analysis needs further clarification. The use of directed acyclic graphs (DAGs) in variable selection could improve transparency and scientific validity, as suggested by previous studies [2]. The definition of chronic diseases in the covariates needs to be further standardized. Specifically, for hypertension, it should be clarified whether the diagnosis is based on multiple measurements of blood pressure exceeding 140/90 mmHg or on the average of three measurements taken on non-simultaneous days. Noteworthy, the study did not consider a number of important potential confounders that could have biased the results. Blood cell counts may be affected by drug use, such as hormonal drugs. In addition to this, the use of angiotensin II receptor blockers (ARB) and angiotensin-converting enzyme inhibitors (ACEI) medications, which have an impact on the progression and outcome of CKD, are included in NHANES. However, this study did not provide statistical information related to medication history. These data could be collected and then statistically adjusted or analyzed in subgroups.

Secondly, the authors used ALI as a nutritional indicator for patients with CKD, which should be tested with several reliable nutritional measures, including the MUST score/NRS-2000, among others. The authors believe that BMI and serum albumin, which make up the ALI, are good indicators of a patient’s nutritional status, but which is not the case. Very low BMI may be associated with cachexia, and obesity may indeed protect patients from death in certain diseases, but it is generally not a good indicator of nutritional status in any case. BMI cannot distinguish between adiposity and muscle mass. Patients with sarcopenia or edema may have a normal or even high BMI but be malnourished [3,4]. Moreover, in this study, there were many patients with renal failure, who often have concomitant fluid retention, which would lead to a falsely high BMI. The same is true for albumin [5], which is only partially affected by nutritional status, with some diseases having a greater impact on albumin levels (e.g., liver disease, infections). And in this study, patients with liver diseases and infections were not excluded. Fluid imbalance also alters albumin concentration, and a portion of the renal failure patients in this study had fluid retention, which led to a decrease in albumin concentration. This means that the specificity/sensitivity of ALI as a predictor of nutritional status in CKD patients is low and remains to be validated. The authors could have explicitly pointed out the limitations of BMI and albumin when discussing the results in order to more fully interpret the clinical significance of ALI.

Finally, as recommended by NHANES [6], creatinine concentrations in the 2017–2018 NHANES data can be corrected for more accurate data. The formula for standard creatinine (mg/dL) is −0.06945 + 1.051* (NHANES 2017–2018 uncorrected serum creatinine, mg/dL). In addition, participants’ ALI levels were tested only once in this study. However, given the dynamic nature of ALI, it is subject to change during follow-up, meaning that the data used in this study do not reflect the long-term nutritional-inflammatory status of the participants. In follow-up studies, ALI should be measured multiple times to capture its dynamic changes to more accurately reflect participants’ nutritional-inflammatory status.

In conclusion, Zhang et al. provide an interesting basis for the use of ALI as a prognostic marker for CKD. However, addressing these limitations through rigorous methodology, validation of markers, and dynamic assessment is essential to enhance the utility of ALI in CKD prognosis. Further characterization in larger prospective studies with diverse populations is also needed. Finally, it could also be explored whether interventions targeting ALI composition (e.g., albumin supplementation, anti-inflammatory therapy) improve the prognosis of CKD patients with low ALI.
